# Involvement of the dopaminergic system in the reward-related behavior of pregabalin

**DOI:** 10.1038/s41598-021-88429-8

**Published:** 2021-05-19

**Authors:** Yusuf S. Althobaiti, Farooq M. Almutairi, Fahad S. Alshehri, Ebtehal Altowairqi, Aliyah M. Marghalani, Amal A. Alghorabi, Walaa F. Alsanie, Ahmed Gaber, Hashem O. Alsaab, Atiah H. Almalki, Alqassem Y. Hakami, Turki Alkhalifa, Ahmad D. Almalki, Ana M. G. Hardy, Zahoor A. Shah

**Affiliations:** 1 Department of Pharmacology and Toxicology, College of Pharmacy, Taif University, P.O. Box 11099, Taif, 21944 Saudi Arabia; 2 Addiction and Neuroscience Research Unit, Taif University, P.O. Box 11099, Taif, 21944 Saudi Arabia; 3 General Administration for Precursors and Laboratories, General Directorate of Narcotics Control, Ministry of Interior, Riyadh, Saudi Arabia; 4 Deanship of Scientific Research, Taif University, Taif, 21944 Saudi Arabia; 5 Department of Pharmacology and Toxicology, College of Pharmacy, Umm Al-Qura University, Makkah, 21955 Saudi Arabia; 6 Department of Clinical Laboratory Sciences, College of Applied Medical Sciences, Taif University, Taif, 21944 Saudi Arabia; 7 Department of Biology, Faculty of Science, Taif University, Taif, 21944 Saudi Arabia; 8 Department of Pharmaceutics and Pharmaceutical Technology, Taif University, Taif, 21944 Saudi Arabia; 9 Department of Pharmaceutical Chemistry, College of Pharmacy, Taif University, P.O. Box 11099, Taif, 21944 Saudi Arabia; 10 College of Medicine, King Saud Bin Abdulaziz University for Health Sciences, Jeddah, Saudi Arabia; 11 King Abdullah International Medical Research Center, Jeddah, Saudi Arabia; 12 Department of Physiology and Pharmacology, College of Medicine and Life Sciences, University of Toledo, Toledo, OH USA; 13 Department of Medicinal and Biological Chemistry, College of Pharmacy and Pharmaceutical Sciences, University of Toledo, Toledo, OH USA; 14 Department of Clinical Laboratories Sciences, University of Hafar Al-Batin, College of Clinical Laboratories Sciences, Hafar Al-Batin, 39923 Saudi Arabia

**Keywords:** Neuroscience, Reward

## Abstract

There has been an increase in cases of drug addiction and prescription drug abuse worldwide. Recently, pregabalin abuse has been a focus for many healthcare agencies, as highlighted by epidemiological studies. We previously evaluated the possibility of pregabalin abuse using the conditioned place preference (CPP) paradigm. We observed that a 60 mg/kg dose could induce CPP in mice and that pregabalin-rewarding properties were mediated through glutamate neurotransmission. Notably, the dopaminergic reward circuitry is also known to play a crucial role in medication-seeking behavior. Therefore, this study aimed to explore the possible involvement of dopaminergic receptor-1 in pregabalin-induced CPP. Mice were randomly allocated to receive saline or the dopamine-1 receptor antagonist SKF-83566 (0.03 mg/kg, intraperitoneal). After 30 min, the mice received either saline or pregabalin (60 mg/kg) during the conditioning phase. Among the control groups that received saline or SKF-83566, the time spent in the two conditioning chambers was not significantly altered. However, among the pregabalin-treated group, there was a marked increase in the time spent in the drug-paired chamber compared to the time spent in the vehicle-paired chamber. Notably, blocking dopamine-1 receptors with SKF-83566 completely prevented pregabalin-induced place preference, thus demonstrating the engagement of the dopaminergic system in pregabalin-induced reward-related behavior.

## Introduction

Worldwide, the abuse of mind-altering prescription drugs has increased dramatically in recent years^1^. The intake of pregabalin in high doses or in combination with other drugs has a significant addiction risk^2,3^. Moreover, the European Medicines Agency and the European Monitoring Centre for Drugs and Drug Addiction have recently reported a list of several drugs with the potential for abuse, including pregabalin, carfentanil, phenibut, and zopiclone^4^. Pregabalin prescriptions have increased by 150% in the UK within the last 5 years^5^. Moreover, the growing black market, including the online availability of pregabalin without a prescription^6^, indicates the importance of understanding the neurochemical effects behind pregabalin addiction.

Pregabalin is a gamma-aminobutyric acid (GABA) analog that binds strongly to the auxiliary alpha-2 delta subunit of the presynaptic voltage-gated calcium channel receptor to reduce the activation of postsynaptic neurotransmitter release^7–10^. Pregabalin is recommended to treat neuropathic pain, partial epilepsy, and common anxiety disorders^11–13^. Moreover, pregabalin is routinely used off-label for many health conditions, including bipolar disorder, trigeminal neuralgia, restless legs syndrome, and alcohol withdrawal states^14–17^. We previously reported that 60 mg/kg pregabalin tends to cause conditioned place preference (CPP) in mice^18^.

The reinforcing and rewarding properties of a variety of abused drugs are related to the neurotransmission of dopamine, a key driver of the neurobiological modifications in drug addiction^19^. Specifically, most drugs of abuse can elevate the extracellular dopamine levels in the nucleus accumbens (NAc), which is involved in the reward circuitry, motivational drive, and learning facilitation^19^. For instance, studies determined that opiate, methamphetamine, and cocaine administration are correlated with an increase in dopamine efflux from key brain regions in animal models^20–23^. Notably, pregabalin could produce changes in dopamine level similar to other drugs of abuse. Previous findings indicate that the dopamine-1 (D_1_) receptor is an important factor in dopaminergic neurotransmission^24–26^. It is involved in strengthening cognitive performance^27–30^, response control^31^, and reward management^32–34^. Therefore, this study aimed to explore the potential causes behind pregabalin’s reinforcing effects, which we hypothesized could involve dopaminergic system activation through the D_1_ receptors.

## Materials and methods

### Animals

We obtained male BALB/c mice from King Fahad Medical Research Center (Jeddah, SA) with a weight range of 25–35 g. Mice had access to food and water ad libitum at standard conditions, where the temperature and relative humidity were adjusted to 21 °C and 30%, respectively, with a 12-h light/dark cycle. Moreover, before the experiments began, the mice were habituated for seven days. All experiments were carried out in accordance with the Institutional Animal Care and Use Committee of the National Institutes of Health and were approved by the Research Ethics Committee at Taif University (42-0112). All methods are reported in accordance with ARRIVE guidelines.

### Drugs and dosing

We dissolved pregabalin (Jamjoom Pharmaceuticals, Jeddah, SA) and SKF-83566 (SKF; Tocris Bioscience, MO, USA) in 0.9% saline. An SKF dose of 0.03 mg/kg was selected, as several studies have indicated that this dose is safe and effective to use in rodents^35,36^. Moreover, cumulative evidence indicates that an SKF dose of 0.03 mg/kg is sufficient for blocking D_1_ receptors in rodents^35,37,38^. Finally, a dose of 0.03 mg/kg of SKF has been shown to effectively block amphetamine- and scopolamine-induced locomotor stereotypy and hyperlocomotion^39,40^.

### Experiments

#### Apparatus

Briefly, the apparatus was constructed using acrylic and had two identically sized conditioning chambers separated by a removable wall. These conditioning chambers differed in tactile and visual cues as previously reported^18^.

#### Conditioned place preference

**Figure 1:**
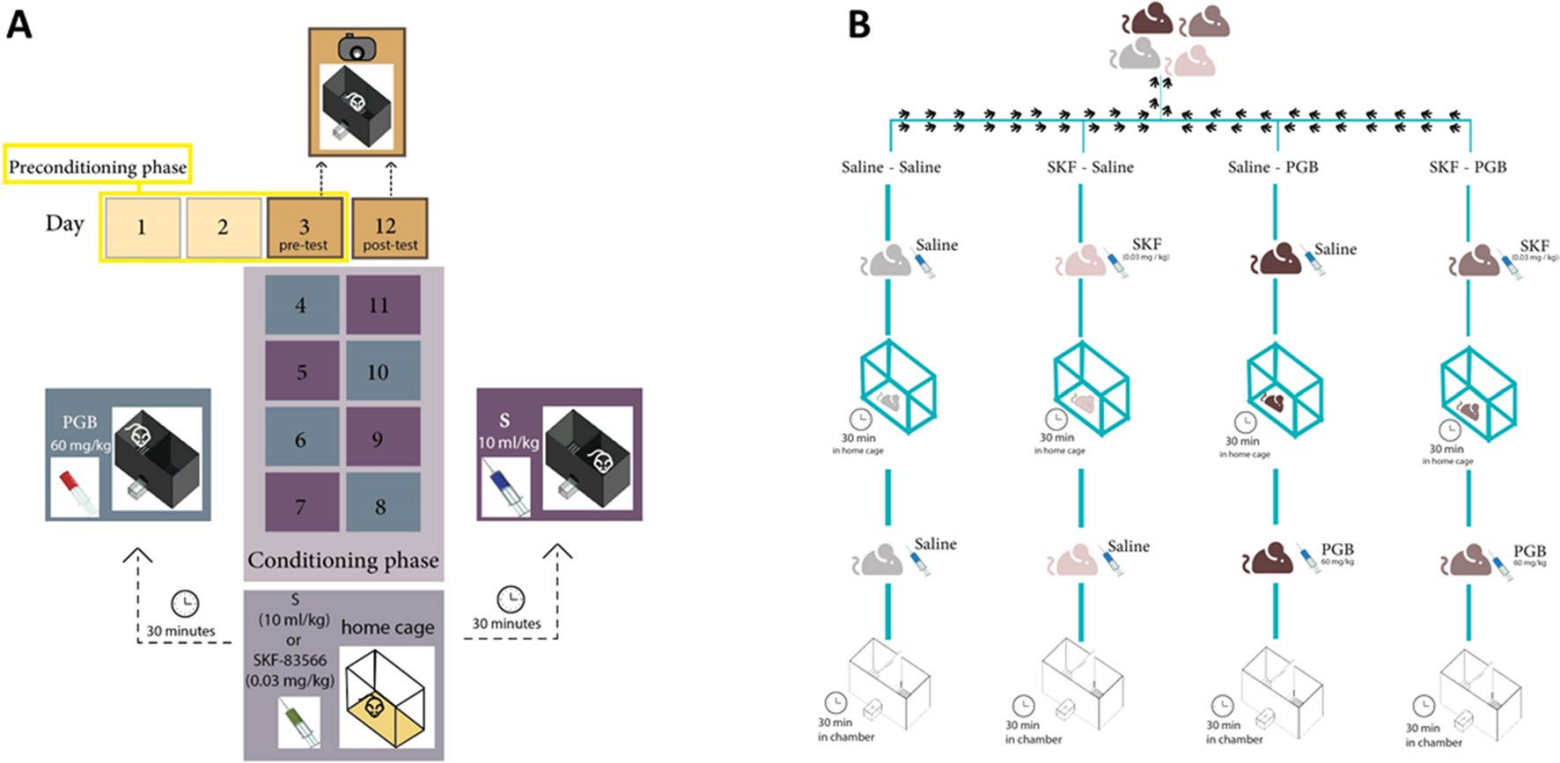
(**A**) Timeline of the CPP experiment. (**B**) The four experimental groups.

We performed the procedure according to the previously reported CPP paradigm, which consisted of two phases: preconditioning and conditioning^18^ (Fig. 1A). During the preconditioning days (days 1, 2, and 3), each mouse was placed in the CPP apparatus with the chamber partition removed, allowing it to move between the two chambers for 30 min, without restriction, to habituate. At the end of day 3, we recorded the time that each mouse spent in each chamber (pretest) using a digital camera, and then analyzed the data using ANY-maze software to determine the baseline values. No mouse had a chamber preference exceeding 67% of the total time of the preconditioning phase.

During the conditioning phase (days 4–11), the mice received pretreatment intraperitoneal injection (i.p.) of saline or the D_1_ receptor antagonist SKF (0.03 mg/kg) 30 min before the administration of saline (10 ml/kg) or pregabalin (PGB) (60 mg/kg, i.p.). Each mouse was administered pregabalin or the vehicle four times every other day for eight conditioning days. Subsequently, we conducted the postconditioning test on day 12, with each mouse being placed in the CPP apparatus without restriction between the chambers for 30 min. Finally, the time spent in each chamber was assessed using the same method that was used to investigate the pretreatment behavior.

#### Procedure

We randomly assigned the mice to one of four groups, as shown in Fig. 1B. Group 1 (Saline-Saline) mice were administered saline (10 ml/kg, i.p.) 30 min before receiving the same dose of saline (10 ml/kg, i.p.) for eight sessions (n = 6). Group 2 (SKF-Saline) mice were administrated SKF (0.03 mg/kg, i.p.) 30 min before receiving saline (10 ml/kg, i.p.) for four sessions (eight sessions in total), with alternating saline sessions during the conditioning phase (n = 8). Group 3 (saline-pregabalin) mice received saline (10 ml/kg, i.p.) 30 min before receiving pregabalin (60 mg/kg) for four sessions (eight sessions in total), with alternating saline sessions during the conditioning phase (n = 6). We selected this dose based on our previous finding that 60 mg/kg pregabalin can induce CPP^18^. Group 4 (SKF-pregabalin) mice received SKF (0.03 mg/kg, i.p.) 30 min before receiving pregabalin (60 mg/kg) for four sessions (eight sessions in total), with alternating saline sessions during the conditioning phase (n = 7). Subsequently, the place preference was assessed after all the conditioning sessions had been completed.

### Statistical analysis

For all CPP behavioral studies, the time that each mouse spent in each chamber pretest and posttest was analyzed using two-way repeated-measures analysis of variance (RM ANOVA). We performed the Newman-Keuls multiple comparisons test using GraphPad Prism. A p-value of < 0.05 was the chosen level of significance.

## Results

### Effects of pretreatment with saline and SKF-83566 on behavioral preference

**Figure 2:**
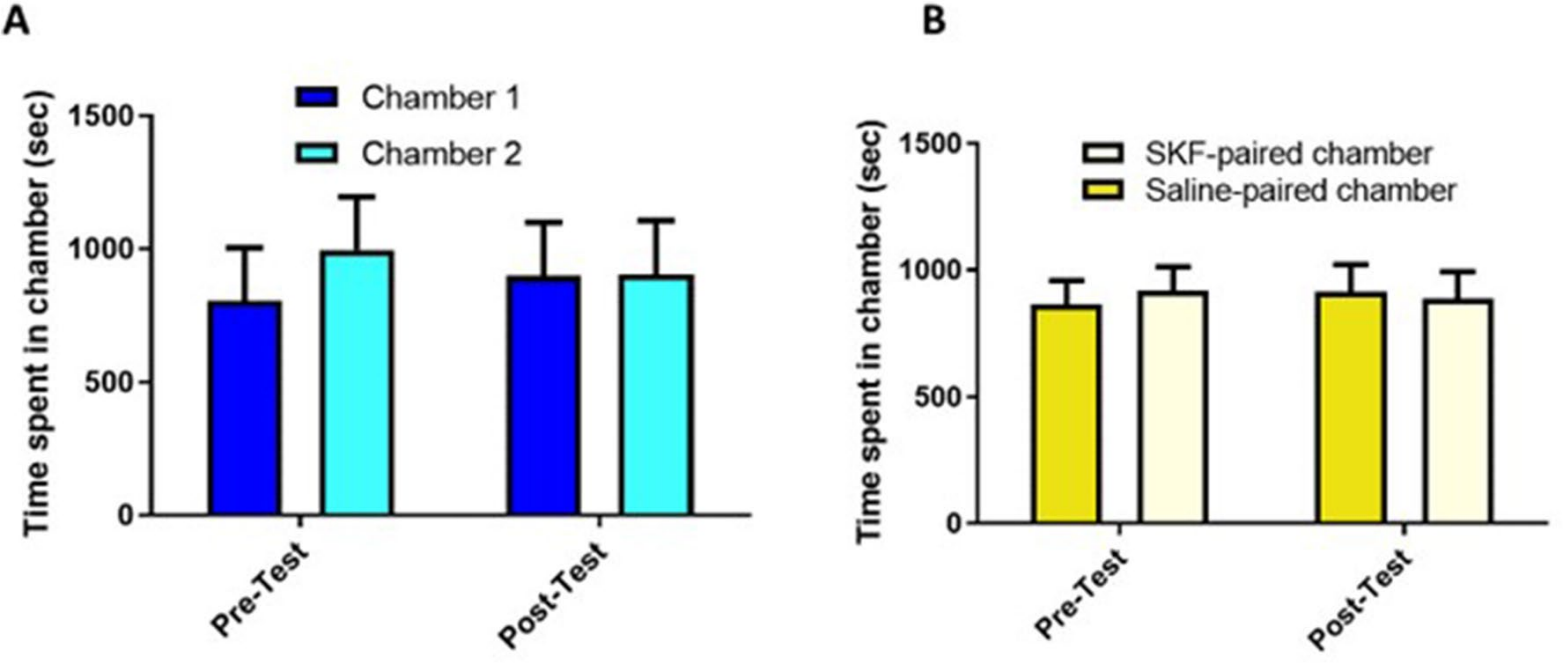
Time spent in the conditioning chambers during the pre and postconditioning tests in the saline-saline (**A**) and SKF-saline (**B**) groups. (**A**) There were no significant changes in the time spent in chamber 1 and 2 during all tested phases (n = 6). (**B**) There were no significant changes in the time spent in the saline-paired chamber compared to the SKF-paired chamber during the pre and postconditioning tests (n = 8). Values are reported as means ± standard error of the mean.

In group 1 (saline–saline), two-way RM ANOVA identified no significant effect on the phase (F (1, 5) = 1.000, p = 0.3632) or chamber (F (1, 5) = 0.07136, p = 0.8000), as well as no significant phase–chamber interactions (F (1, 5) = 0.3981, p = 0.5558) (Fig. 2A). Similarly, in the SKF-saline group (group 2), no significant effect was found on the phase (F (1, 7) = 2.325, p = 0.1712) or chamber (F (1, 7) = 0.007482, p = 0.9335), as well as no significant phase–chamber interactions (F (1, 7) = 0.09655, p = 0.7651) (Fig. 2B).

### Effects of pregabalin and SKF-83566 on pregabalin-induced place preference

**Figure 3:**
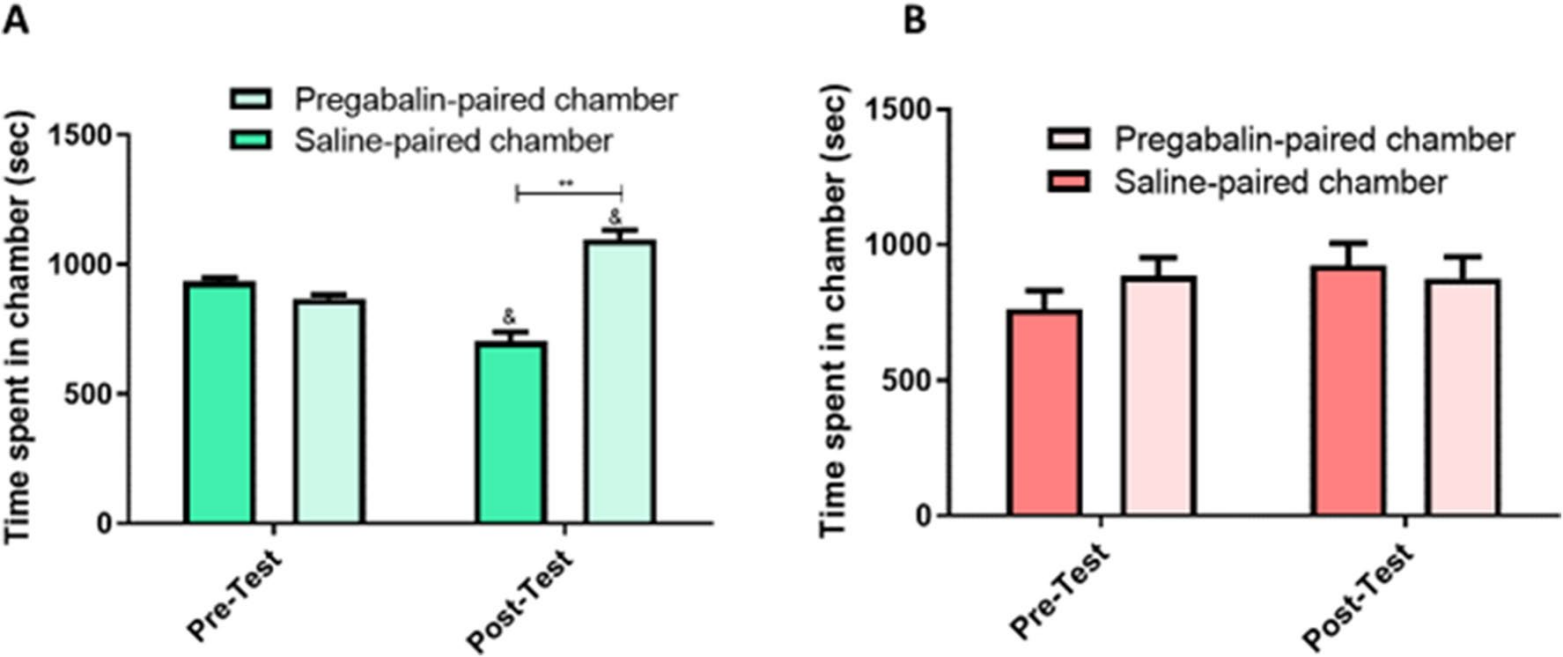
Time spent in the conditioning chambers during the pre and postconditioning tests in the saline-pregabalin (**A**) and SKF-pregabalin (**B**) groups. (**A**) The mice spent significantly more time in the pregabalin-paired chamber during the postconditioning test compared to the preconditioning test. Moreover, there was a significant increase in the time spent in the pregabalin-paired chamber during the postconditioning test compared to that spent in the saline-paired chamber (n = 6). Values are reported as means ± standard error of the mean. (**B**) There was no significant change in the time spent in the conditioning chambers during the pre and postconditioning tests in the SKF-pregabalin group (n = 7). Values are reported as means ± standard error of the mean (**p < 0.01, & *p* < 0.05 compared to the preconditioning test).

In the saline-pregabalin group (group 3), we observed a significant effect on the phase (F (1, 5) = + infinity, p < 0.0001) and chamber (F (1, 5) = 24.90, p = 0.0041), as well as a significant interaction between the phase and chamber (F (1, 5) = 28.55, p = 0.0031). The post hoc analysis revealed a significant increase in the time spent in the pregabalin-paired chamber compared to that spent in the saline-paired chamber during the postconditioning test (p < 0.0100; Fig. 3A). Moreover, there was an increase in the time spent in the pregabalin-paired chamber during the postconditioning test compared to that during the preconditioning test (p < 0.0500). Notably, pretreatment with SKF attenuated the pregabalin-induced CPP. We observed a significant effect on the phase (F (1, 6) = 15,210, p < 0.0001), no significant effect on the chamber (F (1, 6) = 0.08476, P = 0.7807), and no significant interaction between the phase and chamber (F (1, 6) = 1.242, p = 0.3077) (Fig. 3B).

## Discussion

There is empirical proof that the dopamine mechanism plays an essential role in substance-related reward and motivation^41–43^. Moreover, the association between the dopaminergic receptors and the reward signals in the midbrain has revealed that blocking dopamine receptors could attenuate the reward circuitry involved in different drugs of abuse^44–46^. The addictive properties of pregabalin remain controversial; however, several reports have shown an association of pregabalin administration with euphoric effects and abuse potential^3,47^. A previous study demonstrated that pregabalin increased dopamine release in the NAc in a rat model of neuropathic pain^48^. This indicates that exposure to pregabalin could potentiate this connection and subsequently increase dopamine levels. Several reports have shown that SKF is able to interfere with cocaine-evoked dopamine release in vitro, suggesting that this compound may be a potential candidate in attenuating the effects of cocaine in vivo^49^. Moreover, it was reported that pretreatment with SKF blocked amphetamine-induced locomotor stereotypy and hyperlocomotion^39^. Furthermore, SKF blocked the effect of propofol in inducing glutamate neurotransmission in rat midbrain slices via presynaptic D_1_ receptors^50^. Therefore, the D_1_ receptor has been proposed as an important target for testing the behavioral effects related to dopaminergic and glutamatergic neurotransmissions^51,52^. Notably, in the current study, we found that pretreatment with SKF attenuated the reward effects of pregabalin in CPP.

Accumulating evidence indicates that dopamine transport is important for behavioral reward regulation in the NAc^53–55^. Pavlovian conditioning using the CPP model has been known to assess the rewarding effects of drugs^56^ and be dependent on NAc dopaminergic neurotransmission^57^. The mesolimbic dopaminergic pathway, including the ventral tegmental area (VTA) and the NAc, is considered a critical neural region underlying reward and drug-seeking behavior^58^. Activation of D1 receptors, but not D2 receptors, in the NAc is essential for long-term potentiation and positive reinforcement behavior^59^. However, blocking D1 or D2 receptors has shown to impair locomotion and rearing effects associated with dopaminergic neuron inhibition in the NAc core and shell^60^. Several reports have investigated the role of substance abuse in the release of dopamine in rewarding circuits^61^. Importantly, repeated morphine treatment for four doses has been shown to induce CPP in mice^62^. The latter study suggested the development of receptor supersensitivity for postsynaptic dopamine in mice. In confirming this effect, apomorphine (a dopamine agonist) produced stimulated response in ambulatory activity. Moreover, administration of the NMDA antagonist, Mk-801 inhibited the morphine-induced CPP behavior and the development of postsynaptic dopamine receptor supersensitivity. In addition, this effect was observed with other drugs of abuse including cocaine and methamphetamine in mice^63,64^. This is consistent with our findings that blocking D1 receptors attenuated pregabalin-induced CPP. Therefore, pregabalin might induce rewarding effects through the activation of postsynaptic D1 receptors in the NAc. Further studies are needed to examine the effects of pregabalin on dopamine release in the NAc.

Additionally, the glutamatergic system is significantly involved in mediating the drug-seeking effects of several abuse drugs. Drug-seeking behavior has been linked to glutamatergic imbalance in the NAc and downregulation of the glial excitatory amino acid transporter (GLT-1), which is the main regulator of glutamatergic homeostasis in the brain^65–68^. For example, cocaine-seeking behavior has been linked to the downregulation of GLT-1 expression^69^. Furthermore, the disturbance in the glutamatergic system is linked with the spillover of glutamate when the uptake of glutamate by a synapse is decreased, which in turn overactivates the postsynaptic receptors that mediate drug-seeking behavior^70^. Moreover, presynaptic glutamate receptors such as metabotropic glutamate receptors Type 2 (mGlu2/3) have been shown to regulate glutamate release in the NAc and prefrontal cortex and be involved in reward and drug-seeking behavior^71^.

Interestingly, these two systems of dopamine and glutamate have been shown to be interconnected and to influence each other in brain regions. It has been proposed that D_1_ receptors are located in the presynaptic glutamatergic terminal of VTA^72^. Activating D_1_ receptors facilitates the release of glutamate in the VTA^52^. Additionally, it has been mentioned that ethanol-induced spontaneous excitatory postsynaptic currents (sEPSCs) via glutamate alpha-amino-3-hydroxy-5-methylisoxazole-4-propionic acid (AMPA) receptors are suppressed by SKF in the VTA^73,74^. Several studies have shown that ceftriaxone, via the upregulation of GLT-1, can attenuate ethanol intake and relapse in rats^75,76^. The involvement of glutamatergic neurotransmission in pregabalin-induced CPP has been previously reported^18^ and could be due to the activation of presynaptic D_1_ receptors located within glutamatergic synapses. Where, activating the D1 dopamine receptor could augment AMPA receptor transmission as shown in the NAc cell cultures prepared from rat pups^77^. Of note, the interaction between dopamine and glutamate is complex in the NAc. The glutamatergic activation in the VTA has been shown to increase dopaminergic activity and release in the NAc^78,79^. Moreover, glutamate, at presynaptic level in the NAc, can facilitate dopamine release^80,81^. Dopamine can also modulate glutamatergic firings in the NAc that originate from the hippocampus amygdala^82^. Interestingly, this effect has been shown to be mediated through D1 receptors.

Several studies have suggested that repeated dopaminergic activation during behavioral conditioning performs an essential function in the cue stimuli, leading to drug-seeking behavior^83–85^. Reportedly, dopaminergic neurotransmission is associated with glutamate release^86^. In fact, dopamine terminals within the NAc cross on single dendrites with glutamatergic terminals across several key brain regions such as the hippocampus, prefrontal cortex, and amygdala^87^. Furthermore, reports indicate a strong similarity between the activation of the glutamate receptor *N*-methyl-d-aspartate (NMDA) and the D_1_ receptor in drug reward paradigms^88,89^. Moreover, it has been shown that glutamate is released upon dopaminergic neurotransmission in the midbrain region in in vitro and in vivo models^90–95^. For example, in the NAc shell, dopaminergic terminals were found to release glutamate when activated with channelrhodopsin-2^96^. In methamphetamine seeking, both the NAc and dorsomedial prefrontal cortex showed high levels of glutamate and dopamine when analyzed by microdialysis^97^, whereas systemic administration of a D_1_ receptor antagonist (SCH 23390), but not a dopamine receptor-2 antagonist (eticlopride), attenuated methamphetamine seeking^98^. Although standard receptor binding tests have revealed that pregabalin is not bound to the D_1_ receptor^99^, it might increase dopamine levels and glutamate release with the euphoric mental state being achieved. Therefore, the D_1_ receptor antagonist counteracted the D_1_ receptor effects in glutamate release regulation and inhibited the dopamine effects. This could lead to a decrease in glutamate and dopamine release^86,100^. This is in line with our earlier results which confirmed an association between glutamatergic neurotransmission and the rewarding effects of pregabalin^18^. These interconnections between dopamine and glutamate in key brain regions support our previous findings that ceftriaxone, a known regulator of glutamate homeostasis, attenuates pregabalin- induced CPP^18^. Blocking the D1 receptors with SKF in the current study also blocked pregabalin induced CPP. Together, dopaminergic and glutamatergic neurotransmissions in key brain regions might play a significant role in pregabalin-induced CPP. This is consistent with several known drugs of abuse where both dopaminergic and glutamatergic neurotransmissions have been shown to be involved in their rewarding effects^61–68^. Studies are warranted to investigate the neurochemical interactions between dopaminergic and glutamatergic systems in pregabalin induced reward.

The fact that there are no biological studies to confirm these findings is one of the limitations of the present study. Measuring the level of dopamine and glutamate in key brain regions during the conditioning phases, as well as the posttest phase, could provide insights into the mechanisms of pregabalin rewarding properties. A recent study revealed that acute administration of pregabalin did not affect the dopamine level in the NAc^101^. However, the study did not investigate other brain areas such as the NAc subregions (core and shell), prefrontal cortex, or the VTA. Thus, testing only a single area may not be enough to determine the effects of pregabalin on the dopaminergic system as a whole. Another limitation of this study was not assessing the impact of SKF on pregabalin-induced CPP in a dose-dependent manner. This should be considered for examination in future studies. Moreover, it may be worthwhile to assess whether the effect of SKF in blocking pregabalin-induced CPP may also affect other brain neurotransmitters.

A previous in vitro study on human neocortical slices assessed different neurotransmitters and reported that pregabalin modulates acetylcholine, serotonin, and norepinephrine release without changing dopamine release^102^. However, acetylcholine-mediated activation of the D_1_ receptor by SKF-38393 in striatal cells has been shown^103–105^, and this activation was inhibited by SKF^103^. Similarly, SKF appears to partially act against serotonin receptor-1c^106^. Pregabalin interaction with the alpha-2 delta subunit of the calcium channel remains only partially understood. Previous findings regarding pregabalin indicate an essential role of GABAergic neurotransmission in the reward and dependence effects of drugs of abuse^107^. Since pregabalin is a GABA analog, its abuse liability might involve GABA-modulating properties. Specifically, pregabalin administration has been found to slightly increase the extracellular GABA levels in the brain^7,108–110^. Therefore, the weak GABAergic activity of pregabalin may induce GABA-mimetic activity that influences the euphoria and relaxation described by some patients and drug abusers. Thus, future investigations of the effects of SKF on several neurotransmitter systems are needed to understand the mechanistic effect of pregabalin-induced CPP.

In conclusion, findings after pretreatment with SKF indicated that the D_1_ receptors might play a crucial role in the ability of pregabalin to induce behavioral sensitization through the dopamine reward system. However, there is a need for further neurochemical studies to identify similarities in the abuse liability mechanism between pregabalin and other defined addictive drugs.

## Data Availability

The datasets generated during and/or analyzed during the current study are available from the corresponding author on reasonable request.
